# Proteostasis failure and cellular senescence in long‐term cultured postmitotic rat neurons

**DOI:** 10.1111/acel.13071

**Published:** 2019-11-25

**Authors:** Shoma Ishikawa, Fuyuki Ishikawa

**Affiliations:** ^1^ Department of Gene Mechanisms Graduate School of Biostudies Kyoto University Kyoto Japan

**Keywords:** mTOR, postmitotic neurons, proteostasis failure, senescence

## Abstract

Cellular senescence, a stress‐induced irreversible cell cycle arrest, has been defined for mitotic cells and is implicated in aging of replicative tissues. Age‐related functional decline in the brain is often attributed to a failure of protein homeostasis (proteostasis), largely in postmitotic neurons, which accordingly is a process distinct by definition from senescence. It is nevertheless possible that proteostasis failure and cellular senescence have overlapping molecular mechanisms. Here, we identify postmitotic cellular senescence as an adaptive stress response to proteostasis failure. Primary rat hippocampal neurons in long‐term cultures show molecular changes indicative of both senescence (senescence‐associated β‐galactosidase, p16, and loss of lamin B1) and proteostasis failure relevant to Alzheimer's disease. In addition, we demonstrate that the senescent neurons exhibit resistance to stress. Importantly, treatment of the cultures with an mTOR antagonist, protein synthesis inhibitor, or chemical compound that reduces the amount of protein aggregates relieved the proteotoxic stresses as well as the appearance of senescence markers. Our data propose mechanistic insights into the pathophysiological brain aging by establishing senescence as a primary cell‐autonomous neuroprotective response.

## Graphical abstract

Loss of protein homeostasis (proteostasis) is a hallmark of brain aging, yet the adaptive mechanism that contributes to life‐long neuronal preservation is poorly understood. Long‐term cultures of primary post‐mitotic neurons increase a proteotoxic burden and establish cellular senescence, which is alleviated by prolonged treatment of neurons with rapamycin. Post‐mitotic cell senescence is accompanied by stress resilience, suggesting an intrinsic neuroprotective role of senescence.

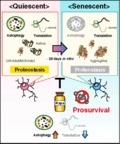

## 1 INTRODUCTION

Most normal somatic cells have a limited capacity for cell division and reach an end stage termed cellular senescence. They can irreversibly cease cell proliferation when challenged by pathophysiological stimuli associated with aging—including telomere erosion, irreparable DNA damage, and hyperactivation of oncogenes (Kuilman, Michaloglou, Mooi, & Peeper, 2010). In addition to the cardinal phenotype of irreversible proliferation arrest, senescence has been characterized by a set of specific markers (Kuilman et al., 2010). These markers have been detected in tissues of aged individuals, such as skin and hematopoietic tissues, suggesting that senescence occurs in vivo (van Deursen, 2014). Notably, it was shown that elimination of senescent cells from normal aged mice ameliorates age‐associated features, suggesting that senescence contributes to the organismal aging process (Baker et al., 2016).

Aging also impairs brain function, such as hippocampus‐dependent cognitive function. The brain is comprised of glial (mitotic) and neuronal (postmitotic) cells. Unlike glial cells, neurons are terminally differentiated, since they have undergone physiological cell cycle arrest. It has been conventionally believed that senescence occurs only in mitotic cells. However, recent studies suggest that it is not always the case (Jurk et al., 2012; Sapieha & Mallette, 2018). For example, postmitotic neurons in rodent hippocampus display a marked increase in senescence‐associated beta‐galactosidase (SA‐β‐gal) activity in long‐term primary cultures (an in vitro model system for studying aging), as well as in aging mouse and rat brains (Geng, Guan, Xu, & Fu, 2010; Piechota et al., 2016). In mouse brain cortex and cerebellum, differentiated neurons undergo a p21‐dependent senescence‐like state, which can be accelerated by deletion of the telomerase RNA gene, aggravating a DNA damage response (DDR) (Jurk et al., 2012). Yet, the underlying mechanisms of postmitotic cell senescence remain elusive. Although brain aging is the process by which neurons progressively lose their plasticity, observed morphological changes in dendritic branching and length, or spine density are very slight, bordering on negligible, in the brain undergoing physiological aging. Moreover, loss of neurons in the entorhinal cortex and hippocampus is not significant in normal aging, while frequently associated with dementia in Alzheimer's disease (AD) (Burke & Barnes, 2006). One current hypothesis for the cause of neuronal loss is proteostasis failure, which is characterized by the accumulation of misfolded proteins and disease‐related toxic proteins (proteotoxic stress). However, proteostasis failure does not always result in neuronal loss, although it impairs hippocampus‐dependent cognitive function in both aged individuals and patients with AD (Kaushik & Cuervo, 2015; Leal, Landau, Bell, & Jagust, 2017). This suggests that proteostasis failure can induce brain aging independently of neuronal loss. Despite extensive studies on proteostasis failure, the adaptive response to proteostasis failure independent of neuronal loss during physiological aging is still largely unknown.

Proteostasis is tightly controlled by the mechanistic/mammalian target of rapamycin (mTOR) pathway, which regulates protein synthesis and degradation upon sensing nutrients and cellular status (Laplante & Sabatini, 2012). It is also thought to be a principle modulator for aging at both the cellular and physiological level. Inhibition of the mTOR pathway improves proteostasis and extends lifespan in a diverse array of organisms (Johnson, Rabinovitch, & Kaeberlein, 2013). Administration of rapamycin, an mTOR complex 1 (mTORC1) inhibitor, ameliorates age‐dependent cognitive deficits in mice (Majumder et al., 2012). Moreover, the mTOR pathway is critical for a transition into senescence in proliferative cells (Leontieva & Blagosklonny, 2010; Young et al., 2009). Although mTOR is involved in both proteostasis failure and senescence, a relationship tying proteostasis failure and senescence to aging in neurons remains to be determined.

Here, we provide evidence that senescence in postmitotic neurons is an adaptive proteotoxic stress response that is likely associated with brain aging. We show that primary neurons subjected to long‐term culture (LTC) express many characteristics of senescence. Such neurons also exhibit changes associated with pathophysiological aging, such as nuclear accumulation of repressor element‐1 silencing transcription factor (REST), also known as neuron‐restrictive silencer factor (NRSF), and proteostasis failure. Our results indicate that downregulation of the mTOR pathway ameliorates senescence in both dividing and postmitotic cells, such as fibroblasts and neurons, respectively, by improving proteostasis. In addition, we found that the senescence‐like neurons are tolerant to stress. Our research uncovers cellular senescence to be a potent neuroprotective mechanism under the AD‐related proteotoxicity and has profound implications for brain aging and neurodegenerative disorders, such as AD.

## 2 RESULTS

### 2.1 Primary rat hippocampal neurons in long‐term culture exhibit senescence‐like phenotypes

**Figure 1:**
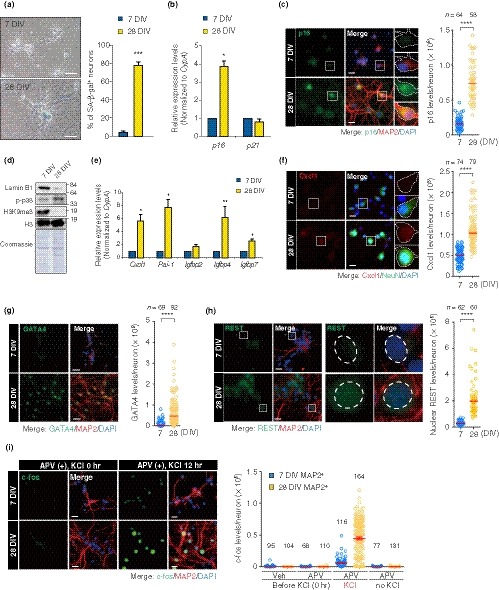
LTC‐PHNs display several features associated with cellular senescence and physiological aging. (a) SA‐β‐gal activity of PHNs was analyzed at 7 DIV and 28 DIV. Scale bar, 40 μm. (b) Abundance of *p16* and *p21* mRNAs at the indicated time points was quantified by RT–qPCR. Expression of the indicated mRNAs was normalized to a housekeeping gene, *CypA*. (c) p16 and MAP2 immunofluorescence performed on PHNs at 7 and 28 DIV. Dashed line demarcates the soma of a representative neuron, staining negative and positive for p16 at 7 and 28 DIV, respectively. Scatter plots showing the signal intensity of p16 in MAP2^+^ neurons, with median indicated. Scale bar, 20 μm. (d) Immunoblotting of the indicated proteins from PHNs at 7 and 28 DIV. (e) SASP genes during LTC of PHNs were analyzed by RT–qPCR. (f) Cxcl1 and NeuN immunofluorescence and quantification of fluorescence intensity of Cxcl1 in NeuN^+^ neurons, with median. A white‐bordered neuronal soma in each enlarged view is Cxcl1^‐^ and Cxcl1^+^ at 7 and 28 DIV, respectively. Scale bar, 20 μm. (g) Immunostaining of GATA4 and MAP2 performed as in (c). Quantification represents GATA4 levels, with median. Scale bar, 30 μm. (h) Nuclear REST detected in MAP2^+^ PHNs by immunostaining. Dashed lines in each enlarged view surround a representative nucleus, staining negative and positive for nuclear REST at 7 and 28 DIV, respectively. A representative quantification of nuclear REST levels is shown with median. Scale bar, 20 μm. (i) Stimulus‐dependent activation of MAP2^+^ PHNs at 7 DIV and 28 DIV was determined by immunostaining of c‐fos (green). Cells were incubated for 24 hr in the presence of 100 μM D‐APV (APV) and were then fixed (0 hr). Alternatively, cells were exposed to 50 mM KCl for an additional 12 hr with APV, followed by fixation (12 hr). Quantification of c‐fos is shown as mean fluorescence intensity (MFI; *n* ≥ 68). Scale bar, 40 μm. The means ± SEM of more than three independent experiments are represented in panels (a), (b), (e), and (i). Unpaired two‐tailed *t* test for (a), (b), and (e); Mann–Whitney U test for (c), (f), (g), and (h) (*p < .05; **p < .005; ***p < .0005; ****p < .0001)

Prolonged culturing induces senescence in various normal mitotic cells, from fibroblasts to epithelial cells, via telomere‐dependent (Herbig, Jobling, Chen, Chen, & Sedivy, 2004) and telomere‐independent mechanisms (Ramirez et al., 2001). It was reported that primary hippocampal neurons obtained from rodent brains exhibited SA‐β‐gal activity following LTC (Geng et al., 2010). To explore this phenomenon in further detail, we set up LTC of primary rat hippocampal neurons (PHNs). Hippocampus‐derived cells were treated with a DNA intercalator AraC from 3 to 4 days in vitro (DIV), thereby enriching the population of neurons positive for neuron‐specific marker NeuN (on average, 76% at 28 DIV, Figure S1A; Beaudoin et al., 2012). To ensure that DNA was no longer replicated in the neurons, we first pulse‐labeled them at 7 DIV with a nucleotide analogue EdU for 24 hr and performed immunofluorescence for NeuN. We detected no EdU‐positive cells that expressed NeuN, indicating that the PHNs at 7 DIV were postmitotic (Figure S1B). We examined SA‐β‐gal activity in the PHNs with typical neuronal morphology and observed greatly elevated number of SA‐β‐gal‐positive neurons at 28 DIV (77.6 ± 4.1%) compared to 7 DIV (4.9 ± 1.3%) (Figure 1a), and SA‐β‐gal‐positive PHNs at 28 DIV exhibited an enlarged morphology (a marked increase in cytoplasmic size, Figure 1a). Of note, the elevated SA‐β‐gal activity was observed irrespective of the treatment of hippocampus‐derived cells with AraC (Figure S1C), suggesting that the senescent phenotypes were not simply due to the AraC treatment. We also found that the PHNs at 28 DIV were positive for the following senescence markers (Kuilman et al., 2010): upregulation of Cdk inhibitor p16 (*Cdkn2a*), but not p21 (*Cdkn1a*) (Figure 1b, c), increased activation of p38 MAP kinase (Phospho‐p38; Figure 1d) and loss of lamin B1 (Figure 1d; Figure S1D, E). Senescent cells ectopically secrete a variety of proteins including inflammatory cytokines—a process termed senescence‐associated secretory phenotype (SASP; Kuilman et al., 2010). The PHNs at 28 DIV showed upregulation of SASP gene expression (Figure 1e), including *Cxcl1*, plasminogen activator inhibitor‐1 (*Pai‐1*), and insulin‐like growth factor‐binding proteins (*Igfbps*). Moreover, we found Cxcl1 proteins to be specifically produced by the PHNs at 28 DIV, but not at 7 DIV and by non‐neuronal cells (Figure 1f; Figure S1F). In mitotic cells, such as fibroblasts, senescence induces a drastic reorganization of chromatin structure, including global loss and/or focal enrichment of heterochromatin marks, such as H3K9me3, associated with dynamic changes of transcriptome signatures essential for the stable and irreversible senescence state (Parry & Narita, 2016). Immunoblotting revealed substantial loss of H3K9me3 in 28 DIV PHNs compared to 7 DIV (Figure 1d). In addition, PHNs at 28 DIV showed increased nuclear size without changes in DNA content (Figure S1G–I), suggestive of the global loss of heterochromatin that is observed in the context of conventional senescence (Parry & Narita, 2016). Persistent activation of DDRs, which is triggered by DNA damage induced by telomere deprotection or DNA hyper‐replication, is responsible for senescence in mitotic cells (Rossiello, Herbig, Longhese, Fumagalli, & d'Adda di Fagagna, 2014). Intriguingly, however, we did not observe an increase in γH2AX immunostaining signals in 28 DIV PHNs compared to 7 DIV (Figure S1J, K), suggesting that there was no significant accumulation of DNA double‐strand breaks (DSBs) in the LTC‐PHNs. Finally, a novel senescence regulator, GATA4 (Kang et al., 2015), accumulated in the 28 DIV PHNs (Figure 1g). Taken together, the postmitotic hippocampal neurons develop an increasing number of hallmarks of senescence over time in vitro.

### 2.2 PHNs in LTC show phenotypes similar to an aging human brain

During neuronal development in humans, transcriptional repressor REST is downregulated to de‐repress neuron‐specific genes. However, in aged brains, REST becomes upregulated to repress apoptosis‐inducing genes, thereby facilitating neuronal cell survival. In contrast, neurons in AD patients lose REST from the nucleus, leading to apoptotic neuronal death (Lu et al., 2014). Immunostaining revealed increased levels of REST in the nuclei of LTC‐PHNs (Figure 1h), consistent with a recent report on primary rat cortical neurons (PCNs) at 30 DIV (Piechota et al., 2016).

Long‐term potentiation (LTP), a long‐lasting enhancement in excitatory synaptic strength, is a prominent form of neuronal plasticity integral to learning and memory. Neuronal activity and plasticity rely on Ca^2+^ influx through two mechanisms, the N‐methyl‐D‐aspartate (NMDA) type of glutamate receptor and the L‐type voltage‐sensitive calcium channel (L‐VSCC). Aging changes the relative dependence of eliciting LTP between these two receptors, which is likely involved in age‐associated cognitive impairment: While NMDA receptors predominantly contribute to memory in young animals, the L‐VSCCs play a major role in aged animals (Disterhoft & Oh, 2006). Thus, we examined the L‐VSCC‐dependent component of neuronal activity in the PHNs through a well‐established method based on activity‐driven transcription of c‐fos following exposure to KCl (stimulation) with D‐APV (D‐(‐)‐2‐amino‐5‐phosphonovaleric acid), an NMDA receptor blocker (Hardingham, Chawla, Cruzalegui, & Bading, 1999). We observed that at 28 DIV, PHNs more strongly induced c‐fos expression 12 hr after stimulation compared to 7 DIV and 14 DIV (Figure 1i; Figure S2), suggesting that the NMDA receptor played a diminishing role over time in neuronal activation caused by KCl, which is consistent with previous studies using the patch‐clump technique in aged hippocampus in rats (Thibault & Landfield, 1996). Taken together, our data suggest that LTC of PHNs leads to a senescence state that shares characteristics with both conventional senescence in mitotic cells and neurons in physiologically aged brain.

### 2.3 Proteostasis is impaired in LTC‐PHNs

**Figure 2:**
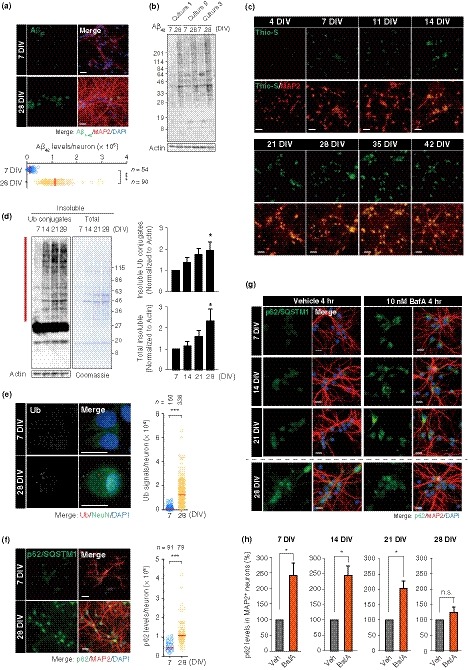
Proteostasis is disrupted in LTC‐PHNs. (a) Immunostaining of Aβ_42_ in MAP2^+^ PHNs and a representative quantification are shown. Scale bar, 40 μm. (b) Immunoblotting of Aβ_42_ with whole‐cell extracts from three independent cultures of PHNs. (c) PHNs at different time points from 4 to 42 DIV were stained with thioflavin S. Scale bar, 40 μm. (d) Immunoblotting for insoluble Ub‐conjugates and Coomassie staining for total insoluble proteins at 7, 14, 21, and 28 DIV. Quantitative data of insoluble Ub‐proteins (top) and insoluble proteins (bottom) normalized to actin from the soluble fraction are shown for the region indicated by the vertical red bar. 7 DIV was set to 1. (e) Immunostaining of Ub‐conjugates and NeuN in PHNs was performed at 7 DIV and 28 DIV. Scale bar, 15 μm. (f) Immunostaining of p62/SQSTM1 and MAP2 performed as in (e). Scale bar, 40 μm. (g) Representative images for autophagic flux analysis by BafA treatment in MAP2^+^ neurons. Scale bar, 20 μm. (h) Quantification of changes in p62 intensity between MAP2^+^ neurons with and without 10 nM BafA. Medians of representative experiments are shown in panels (a), (e), and (f). Data are presented as mean ± SEM of at least three independent experiments in panel (d) and (h). One‐way ANOVA for (d); Mann–Whitney U test for (a), (e), and (f); unpaired two‐tailed *t* test for (h) (*p < .05; **p < .005; ***p < .0001)

Accumulation of intra‐ and extra‐cellular Aβ peptide is a hallmark of aging brains from both healthy individuals and AD patients. Recently, a longitudinal study has revealed that hyperactivity in the hippocampus is associated with increased Aβ deposition and memory deficits during aging (Leal et al., 2017). We observed substantial increases of both intra‐ and extra‐neuronal Aβ_42,_ an aggregate‐prone product derived from amyloid precursor protein (APP) cleavage, and amyloid aggregates within LTC‐PHNs using the specific antibody and amyloid‐binding dye, thioflavin S (Thio‐S) (Figure 2a–c, Figure S3A). Temporal analyses of Thio‐S staining revealed that intra‐neuronal amyloid aggregates began to accumulate by 7 DIV (Figure 2c). Protein aggregates are comprised of misfolded proteins, often conjugated with poly‐ubiquitin (poly‐Ub), as well as disease‐related proteins, such as Aβ_42_, and normally display poor solubility in detergents (Lim & Yue, 2015). As expected, the Triton‐insoluble poly‐Ub conjugates significantly increased over time in the neuronal cultures, which was followed by accumulation of total insoluble proteins (Figure 2d; Figure S3B, C). Consistently, immunostaining analyses revealed higher levels of both Ub‐conjugates and ubiquitin‐binding autophagic adaptor p62 (also known as SQSTM1) in PHNs at 28 DIV compared to the cells at 7 DIV, indicating that misfolded proteins accumulated with time in vitro (Figure 2e, f). To understand how proteostasis is perturbed, we investigated the autophagic flux of the PHNs by immunostaining for p62 following bafilomycin A1 (BafA) (Garcia‐Prat et al., 2016; Mizushima, Yoshimori, & Levine, 2010), an inhibitor of late‐phase autophagy via inactivation of V‐ATPase. We observed robust BafA‐dependent punctate p62 signals at 7 and 14 DIV, indicating an active autophagic flux (Figure 2g, h). In contrast, the BafA‐dependent signal was less pronounced at 21 DIV and was eventually lost at 28 DIV (Figure 2g, h), suggesting that the autophagic activity becomes impaired over time in vitro as recently reported in senescence‐like PCNs at 26 DIV (Moreno‐Blas et al., 2019). Together, these results suggest that proteostasis failure occurred in LTC‐PHNs as evidenced by accumulation of poly‐Ub proteins and amyloid aggregates. It was likely caused via impairment of autophagic clearance during in vitro aging, as is observed in physiological brain aging (Moreno‐Blas et al., 2019; Yang et al., 2014).

### 2.4 AD‐related proteotoxicity causes the senescence response in PHNs

**Figure 3:**
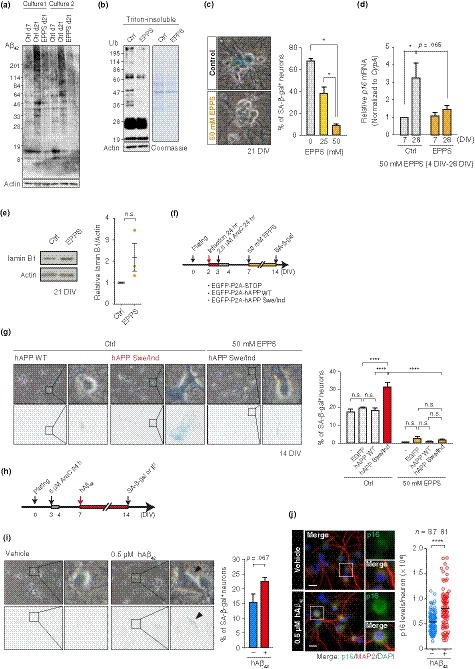
AD‐related proteotoxicity induced senescence features in PHNs. (a) Immunoblotting of Aβ_42_ in total cell extracts from two independent cultures of PHNs that were continuously treated with vehicle (control/Ctrl) or 50 mM EPPS from 4 DIV. (b) Western blot and Coomassie staining of the insoluble fraction from 21 DIV PHNs treated as in (a). Soluble actin is shown as a loading control. (c) SA‐β‐gal activity in 21 DIV PHNs treated as in (a). (d) Quantification of *p16* mRNA by RT–qPCR. (e) Immunoblotting of lamin B1 in Ctrl or EPPS‐treated PHNs, as in (a). (f) Timeline of the experiments in (g). (g) SA‐β‐gal activity in 14 DIV PHNs expressing EGFP, hAPP WT, or hAPP Swe/Ind with or without 50 mM EPPS. (h) Timeline of prolonged exposure to toxic Aβ peptides (0.5 μM) in (i) and (j). (i) SA‐β‐gal activity in 14 DIV PHNs treated as in (h). (j) p16 and MAP2 immunofluorescence performed on PHNs at 14 DIV. Scatter plots showing a representative quantification of p16 levels in MAP2^+^ neurons, with median. Scale bar, 20 μm. The mean ± SEM of at least three independent experiments is presented in panels (c), (d), (e), (g), and (i). One‐way ANOVA for (c); two‐way ANOVA for (d) and (g); unpaired two‐tailed *t* test for (e) and (i); Mann–Whitney U test for (j) (*p ≤ .05; ****p < .0001). n.s., not significant

We then asked whether proteostasis failure contributes to the neuronal senescence. We employed 4‐(2‐hydroxyethyl)‐1‐piperazinepropanesulfonic acid (EPPS), an amyloid‐binding compound that disassembles Aβ aggregates and ameliorates pathological cognitive defects in AD model mice (Kim et al., 2015). Continuous exposure of PHNs to EPPS inhibited the accumulation of high molecular weight Aβ_42_ and insoluble Ub‐proteins (Figure 3a, b), indicating improvement in proteostasis. Interestingly, in this setting, concomitant loss of senescent phenotypes (SA‐β‐gal activity, *p16* elevation, and lamin B1 reduction) was readily apparent in EPPS‐treated PHNs (Figure 3c–e). To further substantiate the direct involvement of the Aβ proteotoxicity, we examined the effects of ectopic expression of human APP with Swedish (KM670/671NL) and Indiana (V717F) familial mutations (hAPP Swe/Ind) on PHNs (Figure 3f, g). The mutant hAPP increased the percentage of PHNs with SA‐β‐gal activity at 14 DIV, whereas neither EGFP nor wild‐type hAPP expressing PHNs accelerated the senescent phenotype (Figure 3g). Importantly, EPPS treatment abrogated elevation of SA‐β‐gal activity by the mutant hAPP (Figure 3g). Moreover, we observed that addition of recombinant Aβ_42_ to cultures of PHNs was sufficient to induce SA‐β‐gal activity and p16 (Figure 3h‐j). Collectively, these results provide evidence that proteostasis failure involving the accumulation of pathological Aβ drives the onset of senescence in PHNs.

### 2.5 Downregulation of the mTOR pathway prevents senescence phenotypes by modulating autophagy and protein synthesis in PHNs

**Figure 4:**
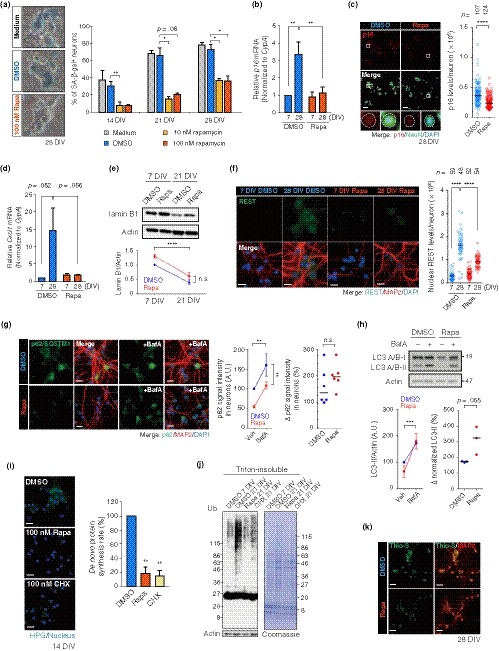
Rapamycin inhibits senescence phenotypes in LTC‐PHNs. (a) SA‐β‐gal staining with PHNs that were continuously exposed to DMSO, 10 or 100 nM rapamycin (Rapa) from 4 DIV until analysis, as indicated. (b), (c) *p16* expression in DMSO and 100 nM Rapa‐treated PHNs was assessed by RT–qPCR (b) and immunostaining (c). A representative quantification of p16 fluorescence intensity in NeuN^+^ neurons at 28 DIV is shown in (c), with the median. Dashed line demarcates a representative soma of a neuron treated with or without Rapa in each enlarged view. Scale bar, 40 μm. (d) With the same conditions as in (b), expression of a SASP gene, *Cxcl1*, was quantified by RT–qPCR. (e) Abundance of lamin B1 protein was analyzed by immunoblotting. (f) Immunostaining of nuclear accumulation of REST (green) in MAP2^+^ PHNs treated with DMSO or Rapa as in (b). A representative quantification of nuclear REST levels is shown, with the median. Scale bar, 15 μm. (g), (h) Autophagic flux analyses using BafA at 21 DIV. 4 hr after BafA treatment, p62 and LC3 were detected by immunostaining (g) and immunoblotting (h), respectively. Relative intensity of p62 in MAP2^+^ neurons was determined by measuring MFI (g, middle). Scale bar, 20 μm. Δp62 signal with and without BafA is also shown (g, right). For LC3, graphs represent levels of LC3‐II normalized to actin (h, left) and its changes with and without BafA (h, right). Horizontal bars indicate median (g, h, right). (i) Global rate of protein synthesis 10 days after culturing with the indicated reagents (at 14 DIV), analyzed by a L‐homopropargylglycine (HPG)‐based fluorescence assay. Fluorescence intensity (green) reflects newly synthesized proteins. Scale bar, 20 μm. (j) Immunoblotting of DMSO, 100 nM Rapa, or 100 nM CHX‐treated PHNs at 7 DIV or 21 DIV with soluble actin as loading control. (k) Thio‐S staining (green) with MAP2 immunofluorescence (red) of 28 DIV PHNs treated with DMSO or Rapa as in (c). Scale bar, 40 μm. Data are presented as the mean ± SEM of at least three independent experiments in panel (a), (b), (d), (e), (g, middle), and (h, left). One‐way ANOVA for (a) and (i); two‐way ANOVA for (b), (d), (e), (f), (g, middle), and (H, left); Mann–Whitney U test for (c); unpaired two‐tailed *t* test for (g, h, right) (*p < .05; **p < .02; ***p < .0005; ****p < .0001)

The mTOR pathway plays pivotal roles in the maintenance of proteostasis via its regulation of autophagy and protein synthesis (Laplante & Sabatini, 2012). mTOR dysregulation contributes to age‐dependent tissue dysfunctions, including cognitive impairment (Johnson et al., 2013; Yang et al., 2014). To gain insights on the role of the mTOR pathway in neuronal senescence, we asked whether rapamycin would protect PHNs from LTC. Chronic exposure to 10 or 100 nM rapamycin from 4 DIV to 14 DIV was sufficient to block the mTOR pathway, as confirmed by reduced phosphorylation of 4E‐BP1 (eukaryotic translation initiation factor 4E‐binding protein) and ribosomal protein S6 (Figure S4). Intriguingly, we found that rapamycin treatment significantly decreased SA‐β‐gal‐positive neurons during LTC (Figure 4a). Moreover, rapamycin inhibited induction of the following senescence phenotypes: p16 elevation, *Cxcl1* upregulation, and lamin B1 loss (Figure 4b–e). It also decreased accumulation of REST in LTC‐PHNs compared to control cells (Figure 4f).

Our observations then raised the question of how rapamycin inhibits senescence phenotypes in PHNs. It has been shown that rapamycin prevents muscle stem cell senescence by restoring autophagy (Garcia‐Prat et al., 2016), which prompted us to examine whether rapamycin elevates autophagy in LTC‐PHNs. Indeed, autophagic flux assays showed a tendency toward greater accumulation of p62 and LC3‐II—an adaptor molecule selective for ubiquitinated aggregates and an autophagosome‐conjugated molecule, respectively—following BafA treatment in rapamycin‐treated PHNs (Figure 4g, h). Of note, prolonged exposure to rapamycin reduced basal levels of p62 in 21 DIV PHNs (Figure 4g, no BafA), suggesting a substantial decrease in misfolded protein aggregates to be degraded by autophagy (Mizushima et al., 2010).

Another mechanism that might ameliorate the neuronal senescence in vitro is inhibition of protein translation. Protein synthesis is regulated by mTORC1, which phosphorylates 4E‐BPs and p70S6 kinases (S6K) for global translational progression (Laplante & Sabatini, 2012). Recent research suggests that reduced protein synthesis is sufficient to slow both cellular and organismal aging (Johnson et al., 2013; Takauji et al., 2016). Therefore, we tested the effects of prolonged exposure to rapamycin on translation by labeling nascent proteins with a methionine analogue and found a significant suppression of global translation in the rapamycin‐treated PHNs (Figure 4i). Because modulation of autophagy and protein synthesis influences proteostasis, we expected that rapamycin could enhance proteostasis in PHNs. Indeed, chronic exposure to 100 nM of rapamycin or cycloheximide (CHX), both of which led to comparable translational inhibition (Figure 4i), significantly decreased the amounts of insoluble (Ub‐) proteins (Figure 4j). Moreover, rapamycin also diminished the Thio‐S‐positive aggregates (Figure 4k). Taken together, these findings demonstrate that rapamycin forestalls the entry of PHNs into a senescent state, possibly by alleviating the AD‐related proteotoxic stress via the modulation of autophagic activity and protein synthesis.

### 2.6 Rapamycin inhibits proteostasis failure and senescence during LTC of PCNs

**Figure 5:**
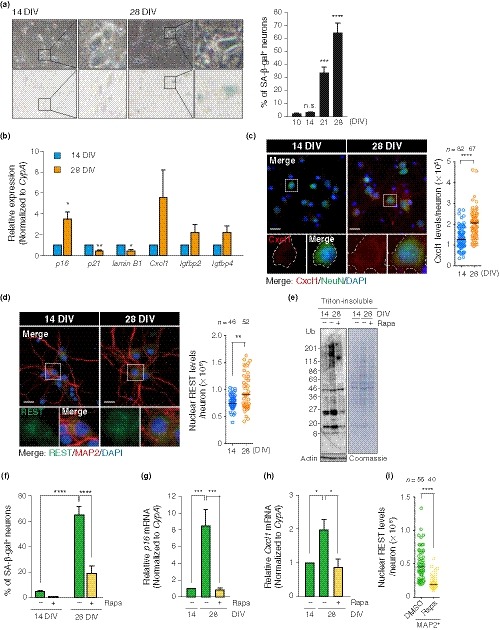
Rapamycin counteracts senescence phenotypes in PCNs. (a) Time‐course analysis of SA‐β‐gal activity of PCNs. (b) Relative changes in expression levels of senescence marker genes measured by RT–qPCR. (c) Immunostaining of Cxcl1 in NeuN^+^ PCNs. A representative quantification of Cxcl1 is shown, with the median. A white‐bordered neuronal soma in each enlarged view shows distinct levels of Cxcl1 protein at 14 and 28 DIV. Scale bar, 20 μm. (d) Immunostaining of nuclear accumulation of REST in MAP2^+^ PCNs. A representative quantification of nuclear REST levels is shown, with the median. Scale bar, 20 μm. (e) Immunoblotting of DMSO or 100 nM rapamycin (Rapa)‐treated PCNs at 14 or 28 DIV with soluble actin as loading control. (f) SA‐β‐gal staining with PCNs that were continuously exposed to DMSO or Rapa from 4 DIV until analysis, as indicated in (e). (g), (h) Expression levels of *p16* and *Cxcl1* mRNA in DMSO and Rapa‐treated PCNs were determined by RT–qPCR. (i) A representative quantification of levels of nuclear REST in MAP2^+^ PCNs at 28 DIV chronically treated with DMSO or Rapa is shown, with the median. The means ± SEM of at least three independent experiments are presented in (a), (b), (f), (g), and (h). One‐way ANOVA in (a), (g), and (h); unpaired two‐tailed *t* test for (b); two‐way ANOVA for (f); Mann–Whitney U test for (c), (d), and (i) (*p < .05; **p < .01; ***p ≤ .002; ****p < .0001). n.s., not significant

We wondered whether the proteotoxic stress‐mediated senescence could be generalized to postmitotic neurons other than hippocampal neurons. Therefore, we verified that PCNs underwent senescence and proteostasis failure during LTC. We confirmed that, as with PHNs, PCNs exhibited the conventional senescent phenotypes—SA‐β‐gal activity (Moreno‐Blas et al., 2019; Piechota et al., 2016), *p16* upregulation, *lamin B1* reduction, and SASP induction (*Cxcl1*, *Igfbp2*, and *Igfbp4*)—as well as elevated levels of nuclear REST (Piechota et al., 2016) by LTC (~ 28 DIV; Figure 5a–d). These cells also exhibited proteostasis failure, as demonstrated by the accumulation of insoluble Ub‐conjugates (Figure 5e).

Next, we examined the effects of rapamycin on the LTC‐PCNs and found a dramatic reduction in insoluble Ub‐conjugates by chronic exposure to rapamycin (Figure 5e). Moreover, consistent with our observations in PHNs (Figure 4), rapamycin inhibited not only senescent phenotypes (SA‐β‐gal activity, *p16*, and *Cxcl1* expressions; Figure 5f–h) but also an age‐related change, nuclear accumulation of REST proteins, in the LTC‐PCNs (Figure 5i). These results further support our findings that inhibition of the mTOR pathway improves proteostasis and counteracts senescence in postmitotic neurons during LTC.

### 2.7 Senescent neurons are resistant to stress

**Figure 6:**
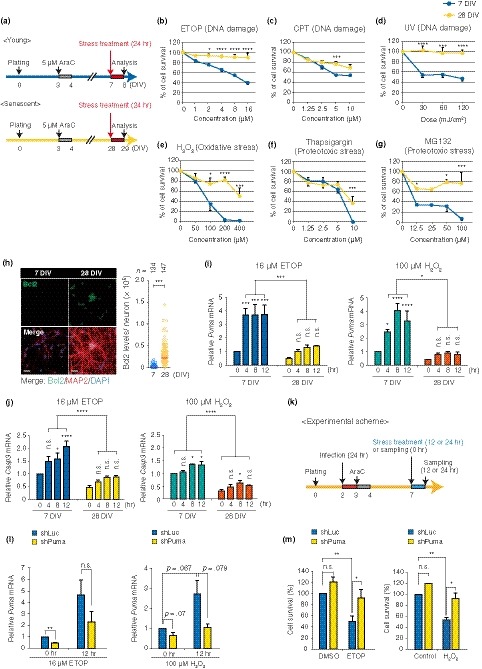
LTC‐induced senescent neurons are resistant to a variety of stresses. (a) Schematic representation of cell survival assay in (b)–(g) (except for UV irradiation in (d), described below). Cell survival of young (blue) and senescent PHNs (yellow) without stress treatment (0 mJ/cm^2^ for UV and 0 μM for others) was defined as 100%. (b)–(g) Cell viability evaluated with a CellTiter‐Glo Kit 24 hr after continuous exposure to indicated stresses (at 8 or 29 DIV). For UV (d), cell viability 48 hr post‐UV irradiation (at 9 or 30 DIV) was measured as well as other stresses. Etoposide (ETOP), camptothecin (CPT). (h) Immunostaining for Bcl2 in 7 and 28 DIV PHNs positive for MAP2. Representative quantified data for Bcl2 intensity in MAP2^+^ neurons are shown (right), with median. Scale bar, 40 μm. (i), (j) Time series analysis of induction of *Puma* (i) and *Casp3* (j) in response to ETOP or H_2_O_2_ in young and senescent PHNs. mRNA levels were normalized to *CypA* expression. (k) Schematic experimental design for knockdown of *Puma* and sample collection. (l) The knockdown efficiency of shPuma before and after exposure to ETOP or H_2_O_2_ was confirmed by RT–qPCR. mRNA levels of *Puma* were normalized to *CypA* expression. (m) Cell survival analysis 24 hr after exposure to stress in control (shLucifierase/shLuc) or Puma depeleted (shPuma) neurons. Cell survival of nonstressed (DMSO or medium change only) neurons expressing shLuc was set to 100%. The means ± SEM of at least three independent experiments are presented in (b)–(g), (i), (j), (l), and (m). Mann–Whitney U test for (h); two‐way ANOVA for other experiments (*p < .05; **p < .01; ***p < .0025; ****p < .0002). n.s., not significant

Postmitotic neurons can be preserved under age‐related proteotoxicity throughout the entire lifespan. Senescent fibroblasts become less sensitive to stress by differentially expressing proteins involved in apoptosis, for example elevating anti‐apoptotic Bcl2, compared to their counterparts (Childs, Baker, Kirkland, Campisi, & Deursen, 2014). These notions led us to examine whether senescence in neurons brought about by LTC imparted stress resistance or not. We first analyzed neuronal survival of young (7 DIV) and senescent (28 DIV) PHNs in vitro after exposure to various stresses: genotoxic stress (etoposide, camptothecin, and UV), oxidative stress (H_2_O_2_), and proteotoxic stress (thapsigargin for ER stress and MG132 for proteasomal inhibition) and (Figure 6a). Intriguingly, for all of the different types of stresses that we tested, the PHNs at 28 DIV showed higher cell viability after stress treatment when compared with neurons at 7 DIV (Figure 6a–g). The stress resistance phenotype at 28 DIV was also correlated with a decrease in the number of cells with condensed or fragmented DNA, indicative of apoptosis, 24 hr after treatment with etoposide or H_2_O_2_ (Figure S5A, B). Moreover, we found that the abundance of Bcl2 was significantly elevated in the PHNs at 28 DIV compared to PHNs at 7 DIV (Figure 6h), whereas the pro‐apoptotic gene known to be a direct target of REST (Lu et al., 2014), p53‐upregulated modulator of apoptosis (*Puma*), showed lower expression levels at 28 DIV compared to 7 DIV (Figure 6i). Expression of *Casp3* and *Puma* was strongly induced by exposure to etoposide or H_2_O_2_ in the PHNs at 7 DIV (Figure 6i, j). Their expression was also induced upon stress in the neurons at 28 DIV, but to a significantly lower extent than in the stressed 7 DIV neurons. The differential expression of *Puma* upon stress between young and senescent neurons was the most conspicuous, which led us to further investigate its role in stress‐induced neuronal death in young neurons. We knocked down *Puma* by short‐hairpin RNA (shRNA) and measured cell survival 24 hr after stress treatment of the PHNs at 7 DIV (Figure 6k–m). Knockdown of *Puma* abolished induction of neuronal death upon etoposide or H_2_O_2_ treatment (Figure 6m), indicating that Puma plays a major role in inducing neuronal apoptosis in young neurons. Finally, the LTC‐PCNs also showed a similar accumulation of Bcl2 and resistance to etoposide as in the PHNs (Figure S6A–C). Taken together, our results demonstrate that senescence state in postmitotic neurons is accompanied by differential expression of apoptosis‐related factors and stress resistance phenotypes, implying the adaptive role of senescence for neuroprotection.

## 3 DISCUSSION

Cellular senescence is a stress‐induced permanent loss of cell division in proliferating cells and is associated with aging. In this study, we have demonstrated that postmitotic rat hippocampal and cortical neurons in vitro exhibit a broad range of classical features of senescence (Kuilman et al., 2010), including SA‐β‐gal activity, p16 upregulation, lamin B1 loss, SASP, and stress‐resistant phenotypes. We also demonstrated that proteostasis failure interconnects with inducing the senescence‐like phenotypes. It has been reported that conventional senescence is caused by proteostasis failure in human primary bronchial epithelial cells (Chong et al., 2018), HUVEC (human umbilical vein endothelial cells; Donnini et al., 2010), normal human fibroblasts (Chondrogianni et al., 2003), and mouse glial cells (Bussian et al., 2018). Furthermore, as has been shown in conventional senescence in human fibroblasts or epithelial cells (Leontieva & Blagosklonny, 2010), we revealed that downregulation of the mTOR pathway by rapamycin mitigates the senescence‐like phenotypes in cultured neurons. Given that the senescence‐like state revealed in rat primary neurons (this study) and conventional senescence share the same causative mechanism and phenotypes, except for proliferation arrest, we argue that the concept of senescence should be extended to postmitotic cells. We suggest that there is a potential causal link between proteostasis failure and senescence in postmitotic neurons in vitro (Figure S7) and suggest that senescence arises in vivo through a similar mechanism.

We also found that the senescent neuronal cells show changes relevant to the pathophysiological aging of the human brain: nuclear REST accumulation and an increase in insoluble poly‐Ub conjugates as well as amyloid‐like aggregates. These phenotypes may be at least in part due to the proteostasis failure (Lu et al., 2014), since reducing proteotoxic stress by rapamycin suppressed the appearance of nuclear REST, suggesting that nuclear REST is a phenotype of neuronal senescence.

It has been proposed that proteostasis failure occurs due to defects in autophagy and/or proteasome‐mediated clearance of misfolded and damaged protein aggregates (Kaushik & Cuervo, 2015). Dysregulation of such protein quality controls has been linked to senescence in proliferative cells (Chondrogianni et al., 2003; Garcia‐Prat et al., 2016; Kang, Lee, Kim, Choi, & Park, 2011). Indeed, the autophagic flux decreased during LTC of PHNs, which was suppressed by inhibiting mTOR with rapamycin. The correlation between autophagic activities and accumulation of misfolded proteins suggests that impaired autophagy is likely to contribute to proteostasis failure in PHNs. Recent findings posit that mitochondria act as an additional hub for maintaining cytosolic proteostasis via degradation of aggregate‐prone proteins (Ruan et al., 2017), along with autophagy and the proteasome. In support of this idea, a previous work and our observations (data not shown) suggest that LTC impairs mitochondrial function in primary neurons (Dong et al., 2011). Further study, however, will be required to identify a mechanistic link between dysregulation of the protein quality control machineries and loss of proteostasis for neuronal senescence.

The pathogenic cascades, starting with Aβ accumulation, appear to begin at least 15–20 years prior to diagnosis of symptomatic stages of AD (i.e., mild cognitive impairment (MCI) and dementia), wherein irreversible loss of neurons and their synaptic connections in the hippocampus and entorhinal cortex occur. One remaining paradox is how the hippocampal and cortical neurons can be preserved at the asymptomatic stage. It is well established that conventional senescence imparts resistance to various stresses (Childs et al., 2014), such as proteotoxic stress, at least in part, by increased levels of Bcl2. Here, we found that senescent neurons in cultures showed a significant increase in Bcl2 and apoptosis resistance against all types of stress tested (genotoxic, oxidative, and proteotoxic stress). Because oxidative stress and DNA damage have been reported to stem from Aβ cytotoxicity (Behl, Davis, Lesley, & Schubert, 1994; Kruman et al., 2004), the senescent neurons are likely attempting to counteract the AD‐related proteotoxicity. Interestingly, a series of studies have demonstrated that cell cycle reactivation precedes neuronal death upon stress (Kruman et al., 2004) and is frequently observed in the neurodegenerating brain of patients with AD (Greene, Biswas, & Liu, 2004). In line with this scenario, *Puma* and *Casp3*, both of which have been shown to be controlled by E2F1 (Bracken, Ciro, Cocito, & Helin, 2004; Hershko & Ginsberg, 2004), a master regulator of cell cycle progression, decreased in the senescent PHNs (Figure 6i, j). Therefore, it will be of interest to investigate whether a shift of cell cycle state from reversible (quiescence) to irreversible state (senescence) is a mechanistic basis for neuronal preservation by preventing apoptosis under cumulative proteotoxic stress with aging.

In conclusion, we have found that cellular senescence in postmitotic neurons is associated with proteostasis failure, and the mTOR pathway at the core of proteostasis is a key regulatory element involved in establishing senescence. On the basis of our work, with the obvious caveat that none of the experiments have yet been confirmed in vivo, we speculate that beyond acting as an intrinsic tumor suppressor in proliferation‐competent cells, senescence may also serve as a cell‐autonomous safeguard mechanism against proteotoxicity and neurodegeneration in neurons. However, there would be a cost to this neuroprotective mechanism: SASP. The occurrence of senescence may explain why physiologically aged hippocampus is not associated with relentless neuronal cell death as observed in AD. This study opens up previously unexplored avenues for deciphering the pathogenic continuum of AD and developing early therapeutic interventions.

## CONFLICT OF INTEREST

The authors declare no conflict of interest.

## AUTHOR CONTRIBUTIONS

S.I. and F.I. designed experiments. S.I. conducted all experiments and analyzed the data. S.I. and F.I. wrote the manuscript.
