# Supplementary material for: Proteostasis failure and cellular senescence in long‐term cultured postmitotic rat neurons
Source: Aging Cell. 2019 Nov 25;19(1):e13071. doi: 10.1111/acel.13071 (PMC6974705; doi:10.1111/acel.13071)
Supplement: Supplementary file 1 [file ACEL-19-e13071-s001.pdf]

Supporting Information

Proteostasis failure and cellular senescence in long-term cultured post-mitotic rat neurons

Shoma Ishikawa[1] and Fuyuki Ishikawa[1]
[1]Department of Gene Mechanisms, Graduate School of Biostudies, Kyoto University, Kyoto, 606-8501, Japan
Correspondence: fishikaw@lif.kyoto-u.ac.jp

Data S1 Experimental Procedures.
Fig. S1 Senescence and aging brain-associated features in LTC-PHNs.
Fig. S2 Stimulus-dependent neuronal activity during LTC.
Fig. S3 Secreted Aβ and Ub-conjugates in LTC-PHNs.
Fig. S4 Rapamycin inhibits the mTOR pathway in primary neurons.
Fig. S5 LTC-PHNs are resistant to apoptosis.
Fig. S6 Stress resistance phenotypes in LTC-PCNs.
Fig. S7 A model of relations between proteostasis failure and senescence in post-mitotic neurons.

References for Supporting Information

Bartlett, B. J., Isakson, P., Lewerenz, J., Sanchez, H., Kotzebue, R. W., Cumming, R. C., . . . Finley, K. D. (2011). p62, Ref(2)P and ubiquitinated proteins are conserved markers of neuronal aging, aggregate formation and progressive autophagic defects. *Autophagy, 7*(6), 572-583.

Dai, S., Tang, Z., Cao, J., Zhou, W., Li, H., Sampson, S., & Dai, C. (2015). Suppression of the HSF1-mediated proteotoxic stress response by the metabolic stress sensor AMPK. *EMBO J,* 34(3), 275-293. doi: 10.15252/embj.201489062.

Garcia-Prat, L., Martinez-Vicente, M., Perdiguero, E., Ortet, L., Rodriguez-Ubreva, J., Rebollo, E., . . . Munoz-Canoves, P. (2016). Autophagy maintains stemness by preventing senescence. *Nature, 529*(7584), 37-42. doi:10.1038/nature16187

Young-Pearse, T. L., Bai, J., Chang, R., Zheng, J. B., LoTurco, J. J., & Selkoe, D. J. (2007). A critical function for beta-amyloid precursor protein in neuronal migration revealed by in utero RNA interference. *J Neurosci*, 27(52), 14459-14469.

Experimental Procedures

Rat hippocampal and cortical neuronal cultures

Dissociated hippocampal or cortical neurons from E18 Wister/ST rats (Japan SLC Inc.) were cultured on plates coated with poly-L-lysine (Sigma-Aldrich) and maintained in neurobasal media (Invitrogen) supplemented with GlutaMAX (Thermo Fischer Scientific), penicillin/streptomycin, and B27 (Thermo Fischer Scientific). Cultured primary hippocampal neurons at 3 DIV were treated with 5 μM AraC (Wako) for 24 h, while cortical neurons at 2 DIV were exposed to 2.5 μM AraC for 36 h. Half of the media was replaced twice every week. For continuous exposure to reagents, cells were treated with rapamycin (Tokyo Chemical Industry), EPPS (Sigma-Aldrich), or CHX (Sigma-Aldrich), from 4 DIV until the respective analyses at the indicated time points. Reagents were freshly added to the media each time that the media was changed.

Chemicals and antibodies

Bleomycin was purchased from Sigma-Aldrich. H_2O_2 was from Wako. Recombinant human amyloid beta 42 protein was from Peptide Institute Inc. (Cat# 4349-V). Anti-actin (Millipore, MAB1501R), anti-p16 (Santa Cruz, sc468; Abcam, ab54210), anti-LaminB1 (Abcam, ab16048), anti-phospho-p38 (Promega, V1211), H3K9me3 (Abcam, 8898), anti-H3 (Abcam, ab1791), anti-Cxcl1 (R&D systems, MAB515), anti-GATA4 (Santa Cruz, sc9053), anti-c-fos (Santa Cruz, sc52), anti-γH2AX (Millipore, 05-636), anti-beta Amyloid 1-42 (Abcam, ab10148), anti-Ub (FK2) (Nippon Bio-test Labo., MFK004), anti-K48-linkage specific poly-Ub (Cell Signaling Technology (CST), 8081), anti-K63-linkage specific poly-Ub (CST, 5621), anti-REST (Santa Cruz, sc25398), anti-p62/SQSTM1 (MBL, PM045), anti-LC3 (CST, 4108), anti-4EBP1 (CST, 9452), anti-phospho-4EBP1 T37/T46 (CST, 9459), anti-phospho-S6 S235/236 (R&D Systems, AF3198), anti-Ribosomal protein S6 (Santa Cruz, sc74459), anti-Bcl2 (Novus Biologicals, NB100-56101), anti-NeuN (Millipore, ABN78), and anti-MAP2 (Santa Cruz, sc32791) were from the indicated suppliers, and were used according to the suppliers' recommendations.

SA-β-gal staining

For SA-β-gal staining, cells were fixed with 0.5% glutaraldehyde in PBS at room temperature for 15 min and incubated in staining solution (PBS containing 0.65 mg/mL X-gal [5-bromo-4-chloro-3-indolyl-β-D-galactoside], 5 mM $K_3Fe(CN)_6$, 5 mM $K_4Fe(CN)_6$, and 1 mM $MgCl_2$, pH6.0) at 37°C for 48 h. For quantification, at least two-hundred neurons in each of three and more independent experiments were ecaluated.

Immunoblotting

Cells were lysed by SDS sample buffer containing β-mercaptoethanol. Whole cell extracts were loaded onto 4-14% SDS-PAGE gels and blotted on a PVDF membrane (Millipore). After blocking in 5% skim milk, 3%BSA, or Blocking One (Nacalai) at room temperature, the blots were incubated O/N with specific antibodies, probed with horseradish peroxidase-linked secondary antibodies (GE Health Care) for 1 h, and detected by ECL reagents (GE Health Care).

Detergent-insoluble protein fractionation and quantification

Triton-insoluble fractions were prepared as previously described (Bartlett *et al*., 2011). In brief, cells were extracted in ice-cold lysis buffer (50 mM NaCl, 5 mM EDTA, 0.1%SDS, 1% Triton X-100, 10 mM Tris-HCl (pH7.4), 1 mM Na_3VO_4, 1 x phosphatase inhibitor cocktail, 30 mM β-Glycerophosphate), centrifuged at 14,000 rpm for 10 min, and the supernatants were collected as the soluble fraction. The remaining pellets were extracted with 2% SDS-containing sample buffer (insoluble fraction). The soluble and insoluble fractions were subjected to SDS-PAGE to detect actin and ubiquitin-conjugates with specific antibodies, respectively. Soluble actin was used as a loading control (Dai *et al*., 2015), and relative amounts of actin in each soluble fraction dictated the respective volumes of the insoluble fractions to be loaded. After detection of the ubiquitin conjugates, the PVDF membrane with the blot of the insoluble fraction was rinsed in TNT buffer (20 mM Tris-HCl (pH7.5), 140 mM NaCl, 0.05% Tween 20 (vol/vol)) and further subjected to Coomassie staining to determine total amounts of the detergent-insoluble proteins. The immunoreactive signals and levels of the total insoluble proteins were quantified by using Image J software.

ELISA for measurement of $A\beta_{42}$

Supernatants were collected every seven days after initiating the neuronal cultures and stored at -80°C until use. Secreted $A\beta_{42}$ across the LTC of PHNs was analyzed in accordance with the manufacturer's instructions for the Human/Rat Amyloid (42) ELISA kit from Wako (Wako, Cat. No. 292-64501).

Immunofluorescence

Cells growing on coverslips were fixed with 4% paraformaldehyde (PFA) at room temperature for 15 min, or methanol on ice for 15 min. PFA-fixed cells were permeabilized by 0.5% triton buffer (20 mM HEPES (pH7.4), 50 mM NaCl, 3 mM

$MgCl_2$, 0.3 M sucrose, 0.5% Triton X-100 (vol/vol)) for 5 min. After two rinses with PBS, cells were blocked in PBS containing 0.1% skim milk (wt/vol) and 0.1% bovine serum albumin (wt/vol) for 0.5 to 1 h. After that, cells were incubated with primary antibodies for 1 h at room temperature. After three PBS washes, cells were incubated with secondary antibodies conjugated with Alexa488 or Cy3 for 1 h at room temperature. Nuclei were counterstained with 1 μg/mL 4',6-diamidino-2-phenylindole (DAPI). For Thio-S staining, blocked samples were incubated with 0.025% Thio-S (wt/vol) for 5 min followed by three 5 min washes in 80% ethanol and a 5 min wash in PBS. After that, samples were incubated with anti-MAP2 as a primary antibody and then the appropriate secondary antibody. Fluorescence images were acquired using a high-resolution microscope DeltaVision Elite (GE Health Care) and were processed to projections of z-sections. The obtained images were subsequently composed and edited in softWoRx software, in which the background was reduced by brightness and contrast adjustments applied to the whole images. Fluorescence intensity of selected proteins for MAP2- or NeuN-positive neurons was determined using Image J software and the mean of the relative fluorescence was represented as MFI. To quantify nuclear levels of REST, individual nuclei in MAP2-positive neurons were manually outlined and the mean of relative fluorescence in the reference channel (Alexa Fluor 488) for the specified nuclear region was measured using Image J.

RT-qPCR

Total RNA was prepared from cells using an RNeasy Mini kit (Qiagen) and transcribed into cDNA using AMV reverse transcriptase (Life Sciences) and an oligo-dT primer. RT-qPCR reactions were performed with SYBR Green PCR Master Mix (Applied Biosystems). The primer sets used were as follows: *p16*, 5’-GGGTCACCGACAGGCATAAC-3’ and 5’-CAGAAGTGAAGCCAAGGAGAAA-3’; *p21*, 5’-GACATCTCAGGGCCGAAAAC-3’ and 5’-GAATGAAGGCTAAGGCAGAAGA-3’; *LaminB1*, 5’-GAATTCTCAGGGAGAGGAGGTT-3’ and 5’-TATTGGATGCTCTTGGGGTTC-3’; *Cxcl1*, 5’-AAACCGAAGTCATAGCCACAC-3’ and 5’-GGGACACCCTTTAGCATCTTT-3’; *Pai-1*, 5’-TCAGACAATGGAAGAGCAACA-3’ and 5’-GTCAGTCATGCCCAGCTTCT-3’; *Igfbp2*, 5’-GCGGGTACCTGTGAAAAGAGA-3’ and 5’-TCCACATGGTTCTCCACCAG-3’; *Igfbp4*, 5’-GGTGTATGCACGGAGCTGTC-3’ and 5’-CTGTTGTTGGGATGCTCACTCT-3’; *Igfbp7*, 5’-CATCACCCAGGTCAGCAAAG-3’ and 5’-ACTTCGCAGCTCAAGTACACC-3’; *Casp3,* 5’-

GACCTTACTCGTGAAGAAATTATGG-3’ and 5’-CTCCATGACTTAGAATCACACACAC-3’; *Puma,* 5’-CACTGATGGAGATACGGACTTG-3’ and 5'-GCCTTTCCTGAGATGGTGGT-3’; *CypA*, 5’-TATCTGCACTGCCAAGACTGAGTC-3’ and 5’-CTTCTTGCTGGTCTTGCCATTCC-3’. Transcripts of the *CypA* housekeeping gene were used as endogenous controls to normalize the expression of each unknown target.

Autophagic flux assay

10 nM bafilomycin was used to inhibit autophagy for 4 h at 37°C and to detect accumulation of autophagosomes by immunoblotting of LC-3 and immunofluorescence of p62 (see Garcia-Prat *et al*., 2016).

***De novo* protein synthesis assay**

Newly synthesized proteins were detected with a Click-iT HPG Alexa Fluor 488 Protein Synthesis Assay Kit (Thermo Fischer Scientific) according to the manufacturer’s protocol. In brief, rat primary hippocampal neurons were maintained in Neurobasal media supplemented with B27 in the absence or presence of 100 nM rapamycin or 100 nM CHX from 4 DIV to 14 DIV (i.e., for 10 days). At 14 DIV, the media was replaced with L-methionine-free media containing 50 µM Click-iT-labeled HPG, and the cells were incubated for 30 min. The cells were then rinsed and fixed with 4% PFA for 15 min. Following Click-iT reaction with Alexa Fluor 488 dye, and counterstaining with NuclearMask, images were acquired using DeltaVision Elite, and were analyzed by Image J.

Plasmids

Short-hairpin sequences targeting the following regions were cloned into pSuper plasmid: rat Puma/Bbc3 (1726-TAGATATACTGGAATGAATTTT-1748) and firefly luciferase (5’-TAAGGCTATGAAGAGATAC-3’). The sh-Puma or sh-Luc amplicons were then shuttled into the pCS2-RNAi-IRES-EGFP vector using BamHI and XhoI. To generate an EGFP expressing vector for control experiments, oligonucleotides comprising a P2Asequence (5’-GCCACCAACTTCAGCCTGCTGAAGCAGGCCGGCGACGTGGAGGAGAACCCCGGCCCC-3’) were introduced in place of the NLS sequence in the pLenti-EF1-EGFP-NLS. pCAX APP695 vectors (wild type (WT) and Swedish/Indiana mutant (Swe/Ind)) were obtained from Dennis Selkoe and Tracy Young-Pearse through Addgene (plasmids #30137 and #30145) (Young-Pearse *et al*., 2007). We PCR-amplified the

APP695 WT and Swe/Ind from the pCAX hAPP plasmids to include flanking ClaI and MluI restriction sites, and we then subcloned them into pLenti-EF1-EGFP-P2A-STOP.

Lentiviral infection

293T cells were maintained in DMEM (Nissui) supplemented with 10% FBS (GIBCO), 2 mM L-glutamine (Sigma), 96 U ml^{-1} Penicillin (Sigma), and 72 U ml^{-1} Streptomycin (Sigma). 293T cells were transfected with Lipofectamine 2000 (Invitrogen) for shRNA knockdown or Polyethylenimine Max (Polysciences, Inc.) for cDNA expression with 9 μg DNA following the manufacturer's instructions. Thirty-six hours after transfection, the viral supernatant was collected and concentrated using Amicon Ultra-15 100K centrifugal filters (Millipore), yielding aliquots of lentiviral particles, which were stored at -80°C until use. At 3 DIV, hippocampal neurons were subjected to lentiviral infection for 24 h by replacing half of the medium with fresh Neurobasal B27 medium containing the lentiviral particles. 24 h after infection, the medium was changed to treat the neurons with AraC for an additional 24 h.

Statistical analysis

We conducted two-tailed unpaired t-tests, one-way ANOVA, Two-way ANOVA, and Mann-Whitney tests using GraphPad Prism software. Bonferroni or Tukey's multiple comparisons were used for post-hoc tests following ANOVA.

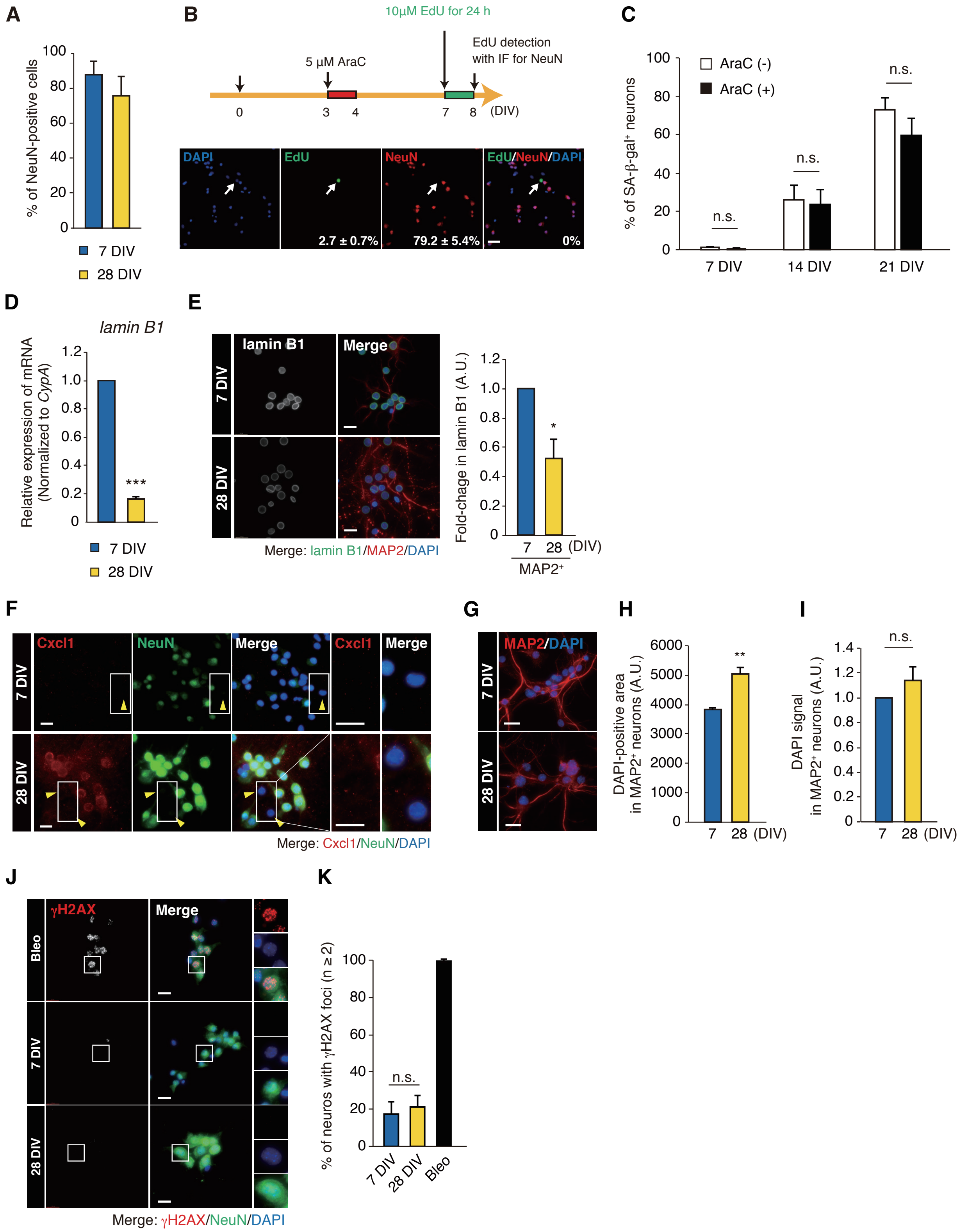

A
% of NeuN-positive cells
100
80
60
40
20
0
7 DIV
28 DIV
B
10μM EdU for 24 h
EdU detection
with IF for NeuN
5 μM AraC
0
3
4
7
8
(DIV)
DAPI
EdU
NeuN
EdU/NeuN/DAPI
2.7 ± 0.7%
79.2 ± 5.4%
0%
C
AraC (-)
AraC (+)
% of SA-β-gal⁺ neurons
n.s.
7 DIV
14 DIV
21 DIV
D
lamin B1
Relative expression of mRNA
(Normalized to CypA)
1.2
1.0
0.8
0.6
0.4
0.2
0
***
E
lamin B1
Merge
7 DIV
28 DIV
Merge: lamin B1/MAP2/DAPI
Fold-chage in lamin B1 (A.U.)
*
7
28
(DIV)
MAP2⁺
F
Cxcl1
NeuN
Merge
Merge: Cxcl1/NeuN/DAPI
G
MAP2/DAPI
H
DAPI-positive area
in MAP2⁺ neurons (A.U.)
6000
5000
4000
3000
2000
1000
**
I
DAPI signal
in MAP2⁺ neurons (A.U.)
1.4
J
γH2AX
Bleo
Merge: γH2AX/NeuN/DAPI
K
% of neuros with γH2AX foci (n ≥ 2)

Figure S1. Senescence and aging brain-associated features in LTC-PHNs

(**A**) The purity of post-mitotic PHNs at the indicated time points was confirmed by IF against neuron-specific marker NeuN. The means ± SD from at least four independent experiments are shown ($n > 500$ cells in each time point of independent experiments).

(**B**) 24 h EdU labeling from 7 to 8 DIV in PHNs. The frequencies of EdU$^+$, NeuN$^+$, and doubly-positive cells are shown at the bottom of each representative image. A white arrow indicates a NeuN-negative cell labeled with EdU (2.7% ± 0.7%). The means ± SEM from three independent experiments are shown (n = 123 to 247/ experiment). Scale bars, 40 μm.

(**C**) SA-β-gal staining of PHNs treated with or without 24 h AraC treatment (solid and white bars, respectively). Data are presented as mean ± SEM from three independent experiments.

(**D**) Relative fold change in *lamin B1* mRNA expression levels between 7 DIV and 28 DIV PHNs was measured by RT-qPCR. Expression of indicated mRNAs was normalized to a housekeeping gene, *CypA*.

(**E**) Immunostaining of lamin B1 protein was performed in PHNs at 7 and 28 DIV. Fold change in the mean for lamin B1 fluorescence was measured with Image J ($n \geq 46$ in each of five independent experiments). Scale bar, 20 μm.

(**F**) Cxcl1 and NeuN immunofluorescence showing SASP in the LTC-PHNs (see also Figure 1F). Yellow arrowheads represent non-neuronal cells negative for NeuN, which appear to no longer produce Cxcl1. Scale bars, 20 μm.

(**G**) Immunostaining of MAP2 protein, counterstained with DAPI in hippocampal neurons at 7 and 28 DIV. Scale bars, 20 μm.

(**H**), (**I**) Nuclear size (H) and contents (I) were determined by measuring DAPI-positive regions and signal intensities in MAP2-positive neurons, as shown in (G), using Image J software ($n \geq 46$ cells per experiment). The means ± SEM from four independent experiments are shown.

(**J**), (**K**) Immunofluorescence of γH2AX (red; left), indicating no DSB accumulation during LTC (28 DIV) of PHNs (J). PHNs at 7 DIV treated with 5 μg/mL of bleomycin for 6 h were used as a positive control for DSB formation. Percentage of neurons positive for NeuN (green) with two or more γH2AX foci is indicated (K). Means ± SD from three independent experiments are shown. Scale bar, 20 μm. Note that at higher magnification the individual γH2AX foci are clearly distinguishable.

One-Way ANOVA for (C); unpaired two-tailed t-test for (D), (E), (H), (I), and (K) (*$p < 0.03$; **$p < 0.01$; ***$p < 0.0005$). n.s., not significant

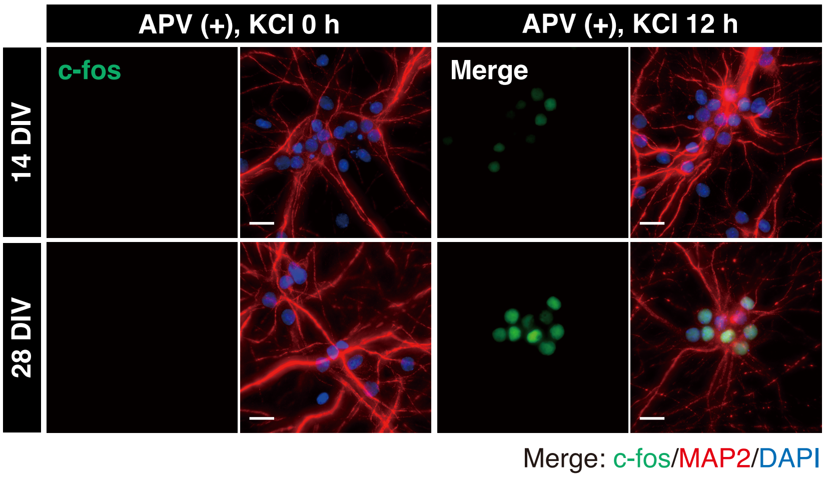

Figure S2. Stimulus-dependent neuronal activity during LTC

Induction of c-fos protein (green) (detected by immunostaining) in MAP2-positive PHNs (red) at 14 DIV and 28 DIV after treatment with KCl as stimulus. Cells were incubated for 24 h in the presence of 100 μM D-APV (APV), and were then fixed (0 h). Alternatively, cells were exposed to 50 mM KCl for an additional 12 h with APV, followed by fixation (12 h). Data are representative of two-independent experiments. Scale bars, 20 μm.

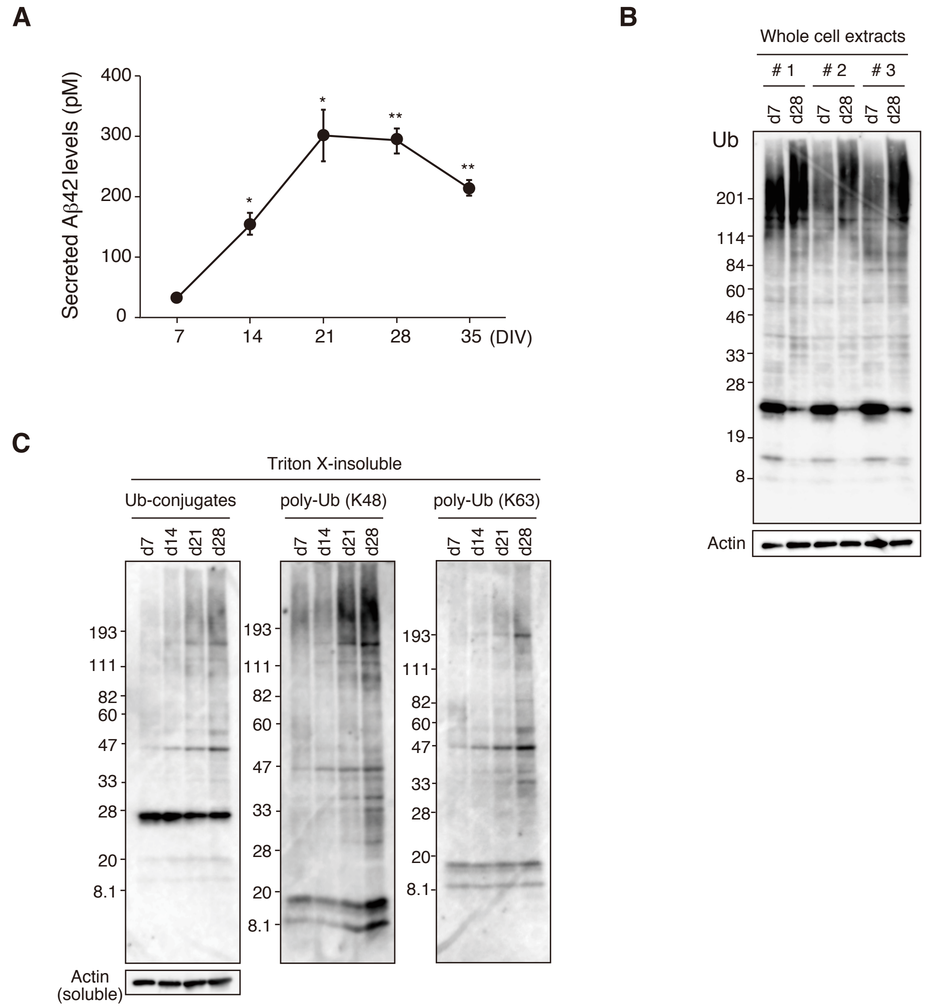

Figure S3. Secreted Aβ and Ub-conjugates in LTC-PHNs

(**A**) Secreted $A\beta_{42}$ within cultured medium from PHNs at the indicated time points was quantified by ELISA. The means ± SEM of four independent primary cultures are shown. One-way ANOVA for statistical analysis (*$p < 0.022$; **$p < 0.006$).

(**B**) Western blot analysis of Ub-proteins with whole cell extracts from three independent cultures of PHNs at 7 and 28 DIV.

(**C**) Immunoblotting of PHN-derived insoluble Ub-proteins (multi (FK2), K48, or K68-linkage specific) at the indicated time points with soluble actin as a loading control. Two independent experiments were performed.

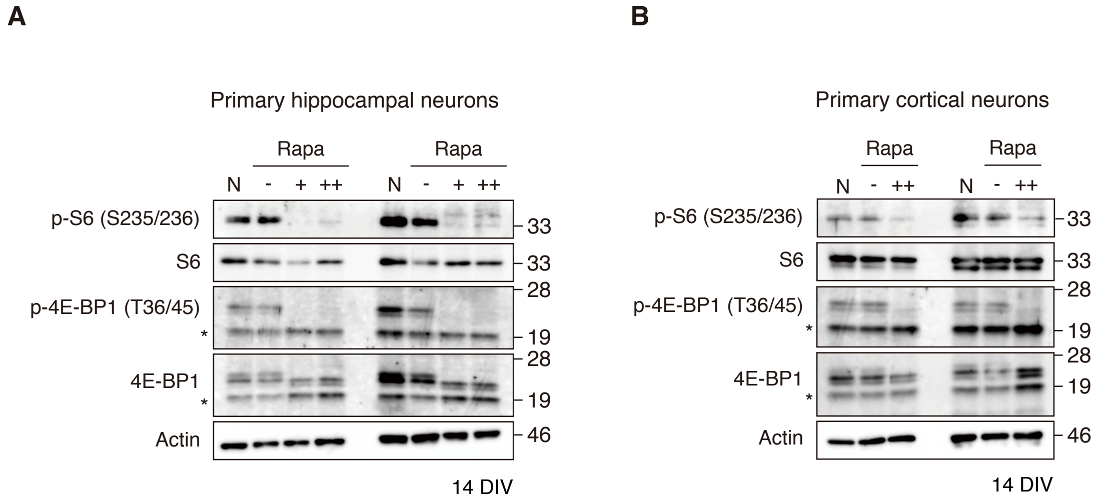

Figure S4. Rapamycin inhibits the mTOR pathway in primary neurons

(**A**), (**B**) Two independent immunoblotting images of phospho-4E-BP1 on Thr36/45 (Thr37/46 in human) and phospho-S6 on Ser235/236 using whole cell extracts obtained from PHNs (A) or PCNs (B) at 14 DIV. The cells were continuously treated with or without DMSO and with rapamycin (Rapa) starting at day 4 (N, no treatment; -, DMSO; +, 10 nM Rapa; ++, 100 nM Rapa). Actin is shown as a loading control. Asterisks represent non-specific signals.

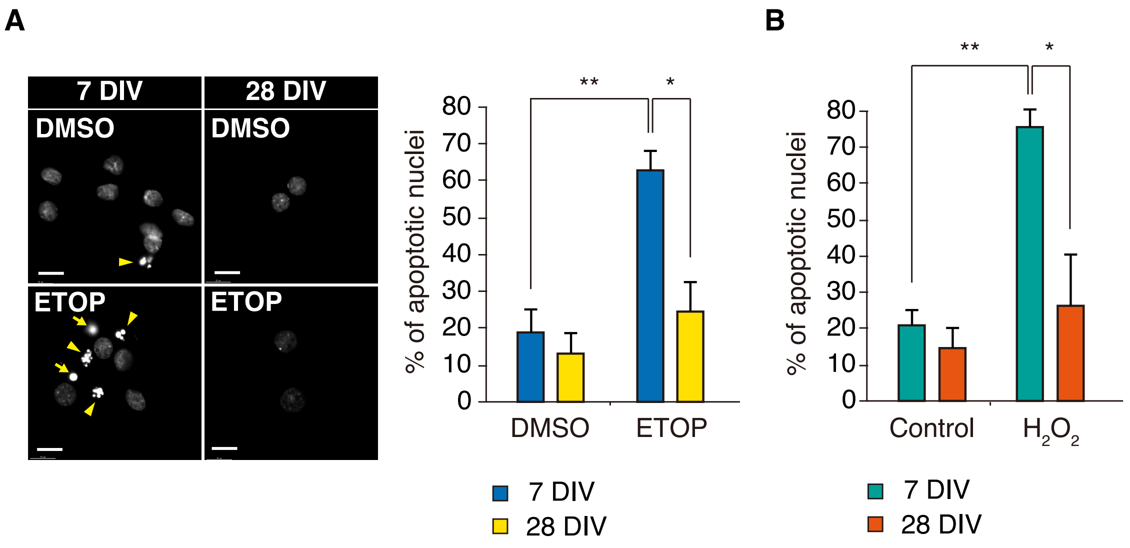

Figure S5. LTC-PHNs are resistant to apoptosis

(**A**), (**B**) Fragmented or condensed nuclei of (apoptotic) neurons that were treated with 16 µM etoposide (A) or 100 µM H_2O_2 (B) at 7 or 28 DIV were counted. 153 to 543 nuclei counterstained by DAPI were analyzed. Stress treatments were performed as shown in Fig. 6A. The means ± SEM of three independent experiments are shown. Arrows indicate condensed nuclei of apoptotic cells, while arrowheads represent fragmented nuclei. Scale bar, 15 µm. Two-way ANOVA for statistical analyses (*$p < 0.04$; **$p < 0.008$).

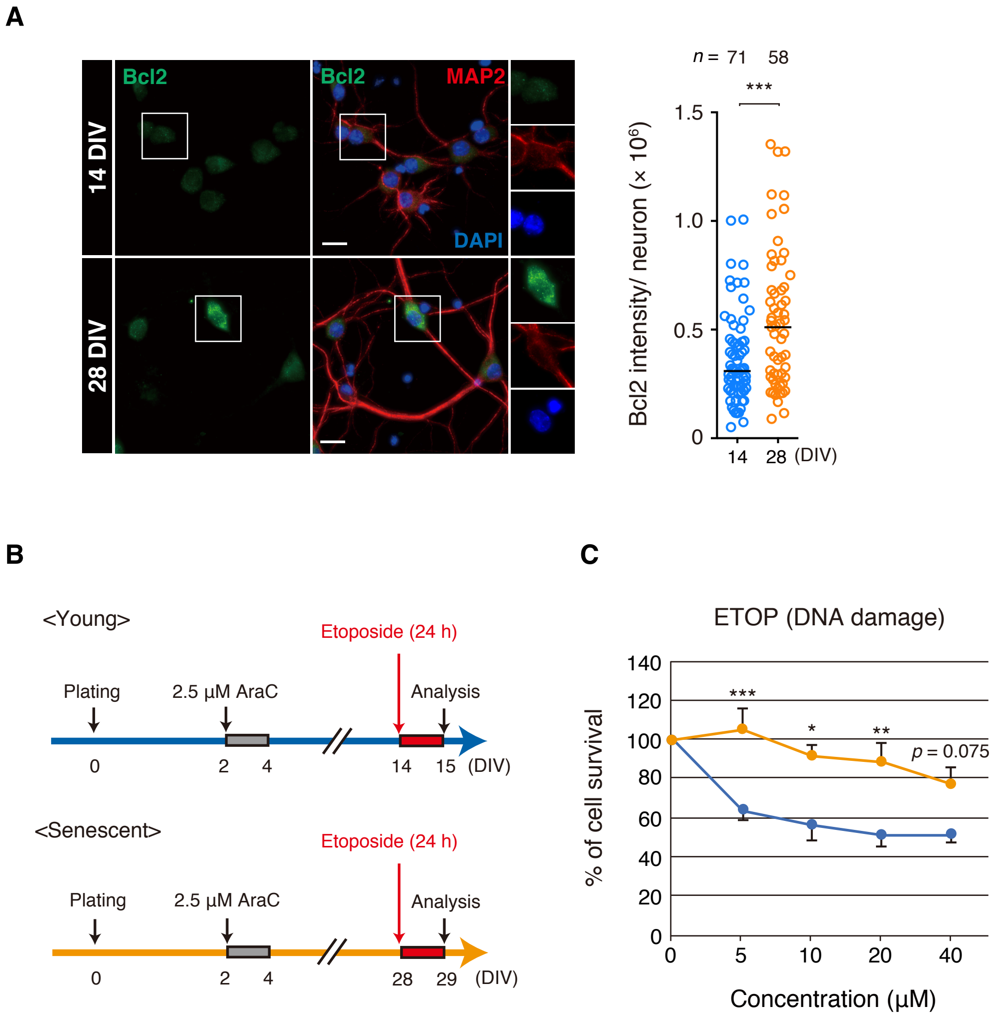

Figure S6. Stress resistance phenotypes in LTC-PCNs

(**A**) Dot blots showing Bcl2 signal intensity in 14 and 28 DIV PCNs positive for MAP2 determined with immunostaining. Nuclei were counterstained with DAPI (blue). Representative quantified data are shown, with median.

(**B**) Schematic outline of the cell survival assay in (C).

(**C**) PCNs at 14 or 28 DIV were exposed to either DMSO (0 μM) or etoposide (ETOP) (5 - 40 μM). Cell survival of young (blue) and senescent cells (orange) without stress treatment (0 μM) was defined as 100%.

Mann-Whitney U-test for (A); Two-way ANOVA for (C) (*p = 0.0056; **p = 0.0032; ***p = 0.0011).

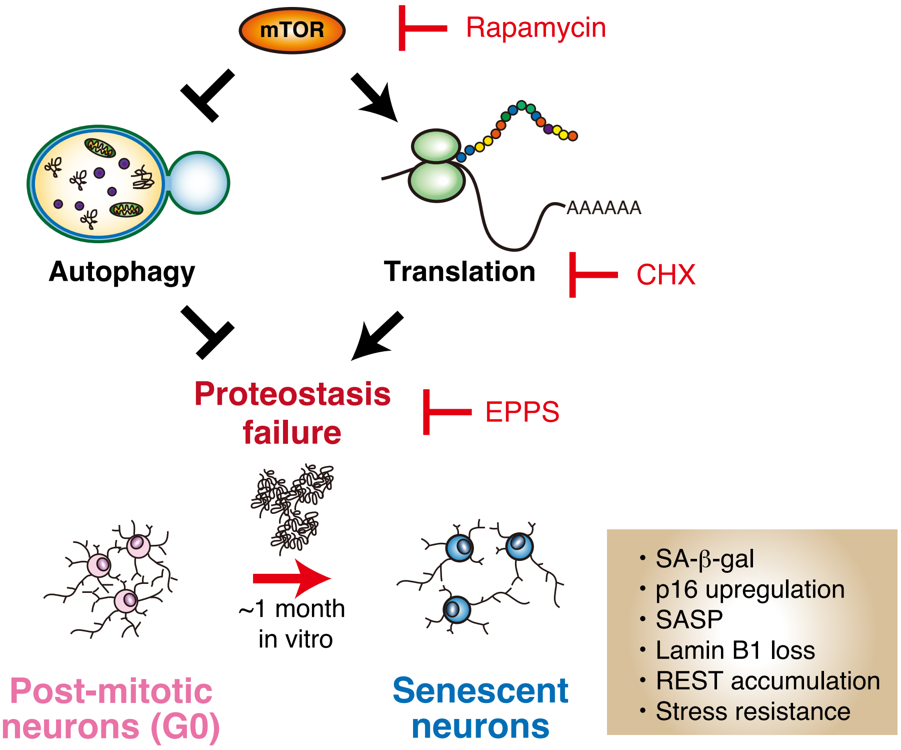

Figure S7. A model of relations between proteostasis failure and senescence in post-mitotic neurons

Post-mitotic hippocampal and cortical neurons display changes associated not only with aging brain, but also with conventional cellular senescence after long-term cultures. Proteostasis failure, possibly due to defective autophagy, is a potential contributor to the age-associated changes. Pharmacological interventions in proteostasis (e.g. rapamycin and EPPS) rescue cellular senescence in post-mitotic neurons. This senescence phenomenon in post-mitotic neurons is accompanied by stress resistance, which may serve as a safeguard against neurodegeneration under age-associated stress, thereby conferring life-lasting survival of neurons during physiological aging.
